# 3D-Printed vs. Heat-Polymerizing and Autopolymerizing Denture Base Acrylic Resins

**DOI:** 10.3390/ma14195781

**Published:** 2021-10-03

**Authors:** Leila Perea-Lowery, Mona Gibreel, Pekka K. Vallittu, Lippo V. Lassila

**Affiliations:** 1 Department of Biomaterials Science, Institute of Dentistry, University of Turku, FI-20520 Turku, Finland; mona.f.gibreel@utu.fi (M.G.); pekval@utu.fi (P.K.V.); liplas@utu.fi (L.V.L.); 2 Turku Clinical Biomaterials Centre-TCBC, Institute of Dentistry, University of Turku, FI-20520 Turku, Finland; 3 Welfare Division, FI-20101 Turku, Finland

**Keywords:** 3D-printing, mechanical properties, post-curing, heat-polymerizing, autopolymerizing

## Abstract

The aim of this work was to investigate the effect of two post-curing methods on the mechanical properties of a 3D-printed denture base material. Additionally, to compare the mechanical properties of that 3D-printed material with those of conventional autopolymerizing and a heat-cured denture base material. A resin for 3D-printing denture base (Imprimo^®^), a heat-polymerizing acrylic resin (Paladon^®^ 65), and an autopolymerizing acrylic resin (Palapress^®^) were investigated. Flexural strength, elastic modulus, fracture toughness, work of fracture, water sorption, and water solubility were evaluated. The 3D-printed test specimens were post-cured using two different units (Imprimo Cure^®^ and Form Cure^®^). The tests were carried out after both dry and 30 days water storage. Data were collected and statistically analyzed. Resin type had a significant effect on the flexural strength, elastic modulus, fracture toughness, and work of fracture (*p* < 0.001). The flexural strength and elastic modulus for the heat-cured polymer were significantly the highest among all investigated groups regardless of the storage condition (*p* < 0.001). The fracture toughness and work of fracture of the 3D-printed material were significantly the lowest (*p* < 0.001). The heat-cured polymer had the lowest significant water solubility (*p* < 0.001). The post-curing method had an impact on the flexural strength of the investigated 3D-printed denture base material. The flexural strength, elastic modulus, fracture toughness, work of fracture of the 3D-printed material were inferior to those of the heat-cured one. Increased post-curing temperature may enhance the flexural properties of resin monomers used for 3D-printing dental appliances.

## 1. Introduction

Complete dentures (CDs) are considered the standard treatment for the rehabilitation of complete edentulism [1,2]. Different techniques, such as dough molding and compression or injection molding, have been utilized for the manufacturing of complete dentures made of polymethyl methacrylate (PMMA)-based resins [3]. However, conventional techniques include a number of laboratory procedures [4]. With the advancement of digital technology, new computer-aided design and computer-aided manufacturing (CAD-CAM)-based techniques have emerged for material processing in dentistry, such as subtractive milling (SM) and additive manufacturing (AM) [5,6]. Thanks to the low technical sensitivity, high accuracy, and method stability of these techniques, dental laboratory operations have become more predictable and time-efficient [6,7]. However, AM or 3D printing is distinguished from SM by its ability to generate multiple complex geometries while minimizing needless waste material [8].

3D-printing technology has been used to create a wide range of dental devices such as surgical guides, provisional crowns, dental splints, and denture bases [5,7,9,10,11]. Due to its superior resolution, precision, quick curing, and low cost, digital light-processing (DLP) is one of the most widely used technologies for 3D printing [12,13]. Printable resins consist of photosensitive thermoset liquid monomers, such as urethane dimethacrylate (UDMA) and triethylene glycol dimethacrylate (TEGDMA), photo-initiators, and additives [7,14,15]. When those monomers are exposed to a suitable light source, a free radical polymerization reaction starts. In that process, terminal aliphatic C=C bonds are broken and converted to primary C–C covalent bonds between methacrylate monomers, causing the material to change from a fluid to a solid state [16].

Usually, the printed components are subjected to a post-curing procedure in an ultraviolet (UV) oven to achieve additional cross-linking of the monomer’s unreacted chemical groups and enhance the mechanical properties [15]. The appropriate post-curing conditions for 3D-printed items are determined by different parameters, such as pigmentation, stability, and resin composition. Additional factors, such as model geometry and object size, may also play a role [17]. For instance, large, complex parts require post-curing units that provide light exposure through a well-balanced light placement or a revolving turntable to ensure consistent curing. The post-curing method has a significant impact on the degree of conversion (DC) of 3D-printed materials [18]. Increasing the DC generally results in improved mechanical characteristics [19], biocompatibility [20], as well as a reduction in residual monomer [21]. This is especially crucial for long-term oral devices that come into contact with soft and hard tissues, such as 3D-printed dentures. Previous studies [10,18,22] found that using different post-curing equipment resulted in considerable variations in the final properties of the printed devices. They reported that light and increased temperature, when involved in the post-curing process, resulted in enhanced mechanical properties and degree of conversion of 3D-printed splints.

Therefore, the aim of the present study was to investigate the effect of two different post-curing methods on the flexural strength, elastic modulus, fracture toughness, work of fracture, water sorption, and water solubility of a 3D-printed denture base material. Additionally, we aimed to compare the mechanical properties of that 3D-printed material with those of conventional autopolymerizing and heat-cured denture base material. The first null hypothesis was that different post-curing methods would not affect the investigated properties of the 3D-printed material, while the second null hypothesis was that all the investigated materials would have similar mechanical properties.

## 2. Materials and Methods

Flexural strength, elastic modulus, fracture toughness, work of fracture, water sorption, and water solubility were investigated for the following denture base materials: a 3D printing (IMPRIMO^®^ LC Denture; Scheu-Dental GmbH, Iserlohn, Germany), a heat-polymerizing (Paladon^®^ 65; Kulzer GmbH, Mitsui Chemicals, Hanau, Germany), and an autopolymerizing acrylic resin (Palapress^®^; Kulzer GmbH, Mitsui Chemicals, Hanau, Germany), which is recommended by the manufacturer for removable partial dentures fabrication (Table 1). IMPRIMO^®^ LC Denture is a light-curing methacrylate-based resin suitable for 3D printers with DLP technology (385 nm). Three test groups were designed for testing as follows: 3D-printed, Palapress, and Paladon. The 3D-printed group was further subdivided into two subgroups according to the post-curing device used: Imprimo Cure and Form Cure (Table 2).

The 3D-printed specimens were virtually designed and then printed horizontally with a DLP 3D-printer (ASIGA MAX™; Scheu-Dental GmbH, Iserlohn, Germany). To remove fluid resin remnants from the test specimens, they were placed in ultrasonic cleaning equipment for 3 min (IMPRIMO^®^ Clean; Scheu-Dental GmbH, Iserlohn, Germany) containing a water-based cleaning agent (IMPRIMO^®^ Cleaning Liquid; Scheu-Dental GmbH, Iserlohn, Germany), and then rinsed with isopropanol for additional 3 min, following the manufacturer’s instructions, in a separate device (Form Wash^®^; Formlabs, Berlin, Germany). Half of the 3D-printed test specimens were post-cured with the Imprimo Cure device (IMPRIMO^®^ Cure; Scheu-Dental GmbH, Iserlohn, Germany), while the other half was post-cured with the Form Cure device (Form cure^®^; Formlabs, Berlin, Germany). The support structures were removed after post-curing using low-speed rotary instruments (5000 rpm). The autopolymerizing and heat-cured acrylic resin test specimens were fabricated according to the manufacturer’ instructions, as described in a previous study [23], and named as Palapress and Paladon groups, respectively. Afterward, the test specimens were finished using silicon carbide grinding papers (800, 1500, and 2000 grit FEPA) and washed with water. Half of the specimens from each group were stored in water for 30 days at 37 °C before testing, while the rest were dry-stored (23 ± 1 °C) for 24 h.

Sixty-four bar-shaped test specimens (10.0 × 65.0 ×3.3 ± 0.2 mm^3^) were tested for flexural strength and elastic modulus (n = 16/group). A static 3-point bending test was conducted in air using a universal testing machine (Model LRX; Lloyds Instruments Ltd., Fareham, UK), at a preload speed of 5 mm/min. PC software (Nexygen 4.0, Lloyd Instruments Ltd., Fareham, UK) was used to record the load-deflection curves and obtain the flexural strength and elastic modulus values. The distance, adjusted to provide support to the test specimens, was 50 mm.

Flexural strength (ơ_f_) and flexural modulus (E_f_) were calculated from the following formula:

ơ_f_ = 3F_m_I/(2bh^2^) (1)

E_f_ = SI^3^/(4bh^3^) (2)

where F_m_ is the applied load (N) at the highest point of the load-deflection curve, I is the span length, b is the width of the test specimens, and h is the thickness of the test specimens.

Additional single-edge notched bend (SENB) test specimens (4.0 × 8.0 × 40.0 mm^3^) were prepared (n = 12/group) to test the fracture toughness (K_Ic_). The specimens were fixed on a flat holder, and a double-faced diamond disk (Komet Dental Gebr. Brassler, Lemgo, Germany) was used to make a 3.0 mm pre-crack in the center of each specimen under water-cooling. The pre-cracks were sharpened by a straight edged razor blade to make a notch with a depth of 100–400 µm. A light microscope (Leica; Leica Microsystem GmbH, Wetzlar, Germany) was used to check the crack length (10× magnification). The test specimens were submitted to a 3-point bending test on a universal testing machine at a crosshead speed of 1.0 mm/min. The specimens were placed on the supports of the test rig (32.0 mm distance) with the notch facing opposite the load plunger. After testing, 3 measurements of the notch length on the fracture surface of each specimen were recorded using the light microscope, and the average was computed and determined as the crack length (a). The *K*_Ic_ was calculated in MPa m^1/2^ using the subsequent mathematical statement [24,25]:

(3) $$K_{\mathrm{Ic}}=\frac{f\;P\;L}{\left({w\;h}^{3/2}\right)}\times \sqrt{10^{-3}}$$

where $f=3x^{\frac{1}{2}}[1.99-x\left(1-x\right)\left(2.15-3.93x+2.7x^{2}\right)]/[2\left(1+2x\right)(1-x{)}^{3/2}]$, x = a/w, P is the maximum load at fracture (N), L corresponds to the span distance (32 mm), h is the height of the specimens in mm, w is the specimen width in mm, and a is the crack length in mm. Then, the total work of fracture (W_f_) was calculated in J/m^2^ as follows [26]:

W_f_ = U/[2 B (H − a)] 1000 (4)

where U is the registered area beneath the load-deflection curve and serves as the energy required to fracture the specimen completely, U = ∫PdΔ in newton millimeters (Nmm), **Δ** is the recorded deflection for load P in newtons, B is the sample width in mm, H is the specimen height in mm, and a is the crack length in mm.

In order to evaluate water sorption and solubility in percentage, forty circular test specimens (n = 10/group) (15.0 ± 0.2 mm in diameter, 2.0 ± 0.2 mm in thickness) were tested. They were kept in an air-drying device involving dried silica at 37 ± 1 °C for 22 h and then at ambient laboratory conditions (23 ± 1 °C) for 2 additional hours. The test specimens were weighed on a scale (XS105; Mettler Toledo, Highstown, NJ, USA) to an accuracy of 0.1 mg to get the values for the initial weight (m_1_). The drying cycle was repeated until the discrepancy among consecutive weight measurements was lower than 0.1 mg. The specimens were submerged in 15 mL of distilled water/sample at 37 °C for 30 days after attaining a constant mass. The weight of the water-stored specimens was evaluated after 60 s of withdrawal from water and thorough drying with absorbent paper at 1, 2, 3, 7, 14, 21, 28, and 30 days. Using the same drying procedure as before, the specimens were reconditioned to a steady mass. The water sorption and solubility rate were calculated adopting the ensuing equations [27,28]:

% Sorption = 100 × (m_2_ − m_3_)/m_1_ (5)

% Solubility = 100 × (m_1_ − m_3_)/m_1_ (6)

where m_1_ is the dry mass (mg) of the sample afterwards storage in an air-drying device for 24 h, m_2_ is the mass (mg) of the specimen after water storage for 30 days, and m_3_ is the steady mass (mg) of the sample subsequent to the second drying cycle.

All data for the evaluated properties were collected and statistically analyzed with a statistical software program (IBM SPSS Statistics, v24; IBM Corp., Armonk, NY, USA). Due to nonhomogeneous variances, Welch’s ANOVA (robust one-way ANOVA) was used to compare data between different groups, while pairwise comparisons of the groups were performed by Dunnet’s T3 post hoc test. Welch *t*-test was used to detect the effect of resin type (conventional or 3D printed), while the effects of post curing method and storage conditions were detected using paired sample *t*-test and Wilcoxon rank-sum test. The significance level was set at *p* < 0.05.

## 3. Results

The type of resin had a significant effect on the flexural strength, elastic modulus, fracture toughness, work of fracture (*p* < 0.001) and water sorption (*p* = 0.004) on the investigated materials. Only flexural strength was significantly affected by the storage condition (*p* = 0.001). The effect of post-curing method was significant on the flexural strength of the 3D-printed specimens (*p* = 0.021).

**Figure 1:**
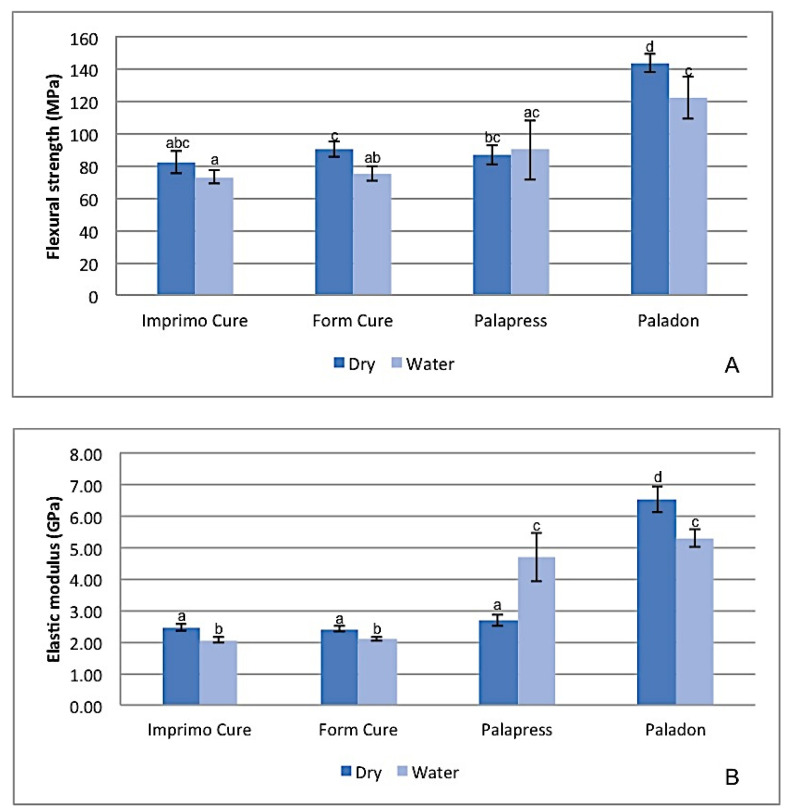
Diagram of flexural strength and elastic modulus mean values for tested groups. Same superscripted lowercase letters illustrate groups/subgroups not statistically significantly different when compared by Dunnett’s T3 post hoc analysis (*p* > 0.05).

The mean values for the flexural strength and elastic modulus of the tested groups are presented in Figure 1. The flexural strength and elastic modulus values for Paladon^®^ were significantly the highest among all investigated groups regardless of the storage condition (*p* < 0.001). However, a significant reduction was seen in both properties after water storage (*p* < 0.05). A non-significant difference (*p* > 0.05) was noticed among the dry samples of the other groups in terms of the flexural strength and elastic modulus values. However, after water storage, the Imprimo Cure^®^ subgroup recorded the lowest strength and modulus, which were significantly lower than Paladon^®^ (*p* < 0.001).

**Figure 2:**
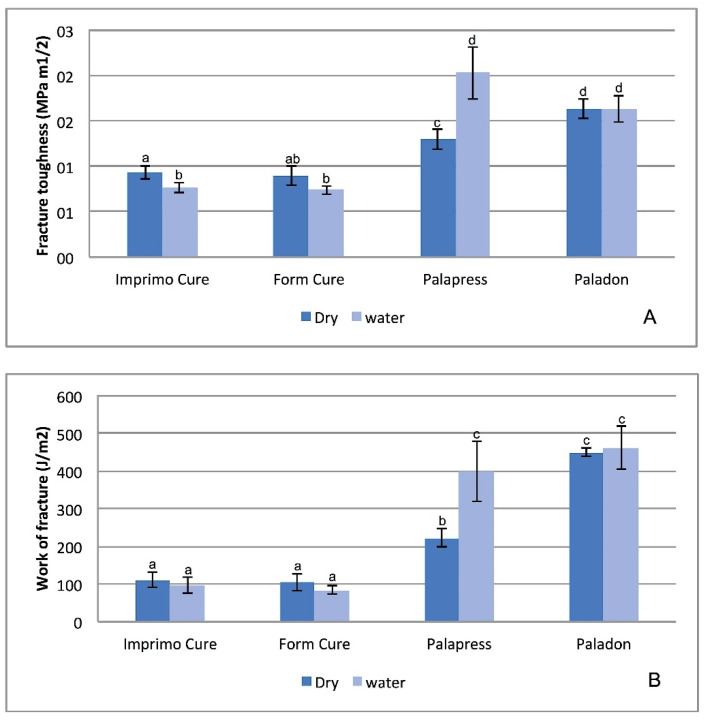
Diagram of fracture toughness (*K*_Ic_) and fracture work (W_f_) mean values for tested groups. Same superscripted lowercase letters show groups/subgroups not statistically significantly different when compared by Dunnett’s T3 post hoc analysis (*p* > 0.05).

Figure 2 shows that the Imprimo Cure and Form Cure subgroups had significantly lower fracture toughness and work of fracture than Paladon and Palapress (*p* < 0.001) regardless of the storage condition. Water storage resulted in a significant increase in fracture toughness and fracture work values for Palapress (*p* < 0.004, *p* = 0.032).

**Figure 3:**
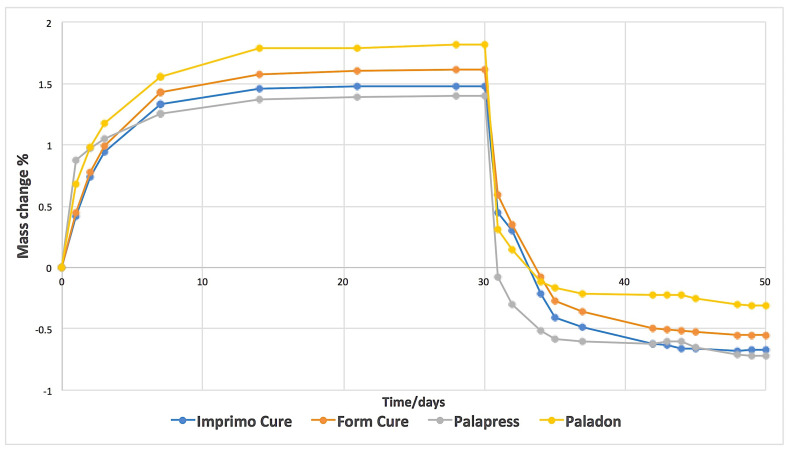
A representative plot of mass change % against time for tested groups/subgroups during water immersion and drying.

Water sorption and solubility values were significantly different between groups (*p* < 0.001) (Table 3). Figure 3 shows a representative plot of mass changes in percentage against time. Water saturation was achieved after 14 days of water immersion. The beginning of the drying process showed fast water loss, which was similar for all the investigated materials. The water sorption values for Imprimo Cure^®^, Form Cure^®^, and Paladon^®^ were statistically non-significant from each other (*p* = 0.167). The water solubility for Paladon^®^ was significantly the lowest among the groups (*p* < 0.001). A statistically significant difference was found between the water solubility values of the Imprimo Cure^®^ and Form Cure^®^ subgroups (*p* < 0.001).

## 4. Discussion

In this in vitro study, the effect of two different post-curing methods on the mechanical properties of a 3D-printed denture base material was investigated. Those investigated mechanical properties were flexural strength, elastic modulus, fracture toughness, work of fracture, and water sorption and solubility. Additionally, a comparison of those mechanical properties was made between conventional and 3D-printed resins. The findings revealed that the post-curing method had a significant effect on the flexural strength of both the dry-stored samples, and the water solubility of the 3D-printed resin material. Furthermore, substantial differences between the characteristics of 3D-printed and conventional denture base materials were found. As a result, the first null hypothesis was partially rejected, while the second hypothesis was totally rejected.

DLP is one of the most advantageous 3D-printing technologies for dental applications due to its quick processing speed, superior resolution, and reasonable cost of the printer and its components [7,14,29]. During printing, the light from a DLP projector delivers energy to polymerize photosensitive materials layer by layer [30].

The flexural strength of a material is defined as the maximum bending stress that can be applied to that material before it yields. Denture bases are prone to fracture when subjected to static or dynamic loading [31]. Therefore, high-flexural-strength values are clinically relevant for reducing the number of denture base fractures. Flexural strength, elastic modulus, fracture toughness, and work of fracture were the lowest for the 3D-printed material. This can be explained by the combination of the reactivity of monomers of 3D-printing resin and the curing condition, which resulted in a lower degree of double-bond conversion when compared to conventional acrylic resins [31,32]. Another cause for the lower mechanical properties could be the weak interlayer bonding between successive printed layers [33,34]. Similarly, Prpic et al. [31], found that 3D-printed denture base materials had lower mechanical properties than CAD-CAM milled and heat-cured ones. However, the 3D-printed denture base material investigated in this study fulfilled the ISO requirements for flexural strength (65 MPa) [35]. Therefore, 3D-printed materials can be considered as an option when fabricating denture bases.

The combined effect of photo and thermal polymerization, as well as the extended post-curing duration within the Form Cure^®^ device, may elucidate the post-curing method’s significant effect on the flexural strength and water solubility of the 3D-printed material. Light intensity and temperature, for instance, have a considerable effect on the degree of double bond conversion and polymer characteristics [36,37,38]. Increases in the resin monomer temperature have been associated with a reduction in its viscosity and an increase in free radical movement. As a result, polymer chains with a higher degree of cross-linking are formed [15,36,38,39]. Likewise, Alsandy et al. [7], investigated the influence of extra light and heat polymerization on the mechanical and physical characteristics of a UDMA-based 3D-printed material used for crown fabrications. They found that heat curing decreased their residual monomer content and enhanced their mechanical characteristics. Additionally, increasing the post-curing temperature and duration resulted in significant enhancements in the flexural properties and biocompatibility of a 3D-printed denture teeth material [15]. Another finding from the same study [15] was that a longer post-curing time at a low temperature provides comparable outcomes to a shorter post-curing time at a higher post-curing temperature.

Water uptake by resin materials is a diffusion-controlled process, which occurs either through its penetration into empty space such as micro-voids, or by a particular molecular interaction [40,41]. The latter depends on resin polarity, which is the number of polar sites that are accessible for hydrogen bonding with water [42]. Water–polymer chain interaction may cause a reduction in the material’s strength, minor chemical degradation, and elution of residual monomers [42]. As a result, water sorption and solubility are crucial indices while assessing denture base durability, since they measure the material resistance to the surrounding oral fluids [25,43]. The 3D-printed material showed a similar tendency toward water sorption as the conventional ones. However, its water solubility was higher than the heat-cured material. This might be attributed to the fact that heat-cured polymers are processed at a higher temperature for a longer duration, resulting in reduced water sorption, solubility, and residual monomer concentration, as stated in previous reports [44,45,46,47,48]. Additionally, the differences in chemical composition between 3D-printed and conventional resin materials must be considered, since the chemical composition of the resin material played a role in its water sorption and solubility [43,49,50].

According to the findings of this study, clinicians should consider the differences in mechanical characteristics between conventional and the 3D-printed materials used for denture base construction. Further improvements in the 3D-printed resin materials’ properties by composition modification or reinforcement are still needed. The correct selection of post-curing method could be an option for improvement.

This study was limited by the investigation of only one type of 3D-printed denture base material and only two post-curing units. Furthermore, the produced specimens did not replicate the denture configuration. Further research investigating various 3D-printed materials and post-curing methods is recommended.

## 5. Conclusions

According to the findings of this study, the following can be concluded:

1. The post-curing method has an impact on the flexural strength of the investigated 3D-printed denture base material. Increased the post-curing temperature may enhance the flexural properties of resin monomers used for the 3D-printing of dental appliances.
2. Higher water solubility and inferior mechanical properties were found on the 3D-printed material when compared to the heat-cured one. The high temperature and extended processing time used for the heat-cured polymers might be attributed to their reduced water sorption and solubility.

## Figures and Tables

**Table 1:** Name, manufacturer, type, chemical composition, and processing method of evaluated denture base resin materials.

Brand Name	Manufacturer	Type	Chemical Composition According to Manufacturer	Processing Method
IMPRIMO^®^ LC Denture	Scheu-Dental GmbH	Methacrylate-based	95% Esterification products of 4,4′-isopropylidenediphenol, ethoxylated and 2-methylprop-2-enoic acid <2% Diphenyl-(2,4,6-trimethylmenzoyl) phosphinoxide (photo initiator)	3D printing: photopolymerization
Palapress^®^	Kulzer GmbH	PMMA-based	Liquid: methylmethacrylate (>90%); tetramethylene dimethacrylate (0–5%); 2-(2H-Benzotriazol-2-yl)-4-methylphenol (<1%), N,N-dimethyl-p-toluidine (<1%) Powder: polymethylmethacrylate (>95%); Bis(p-Chlorbenzoyl) peroxide (0–5%)	Conventional: autopolymerization
Paladon^®^ 65	Kulzer GmbH	PMMA-based	Liquid: methylmethacrylate (>90%), BDMA (0–5%) Powder: Methacrylate copolymonomers (0–5%), BPO < 1%	Conventional: heat-polymerization

**Table 2:** Characteristics of investigated post-curing devices.

Brand	Technology	Duration	Working Pressure	Working Temperature	Wavelength	Manufacturer
Imprimo^®^ Cure	UV LED, nitrogen gas atmosphere	10 min	180 kPa	_	365 and 405 nm	Scheu-Dental GmbH
Form Cure^®^	LED	30 min	_	60 °C	405 nm	Formlabs

**Table 3:** Mean values of water sorption and water solubility % of tested groups.

Group	Subgroup	Water Sorption % (Mean ± SD)	Water Solubility % (Mean ± SD)
3D-printed	Imprimo Cure^®^	2.2 ± 0.01 ^a^	0.67 ± 0.024 ^a^
	Form cure^®^	2.2 ± 0.008 ^a^	0.55 ± 0.027 ^b^
Palapress^®^	-	2.1 ± 0.02 ^b^	0.72 ± 0.096 ^a^
Paladon^®^	-	2.1 ± 0.06 ^ab^	0.32 ± 0.024 ^c^
*p*-value (Welch’s ANOVA)		<0.001	<0.001

Note: Same superscripted lowercase letters show groups/subgroups not statistically different when compared by Dunnett’s T3 post hoc analysis (*p* > 0.05).

## Data Availability

Not applicable.
